# Advances in the use of exosomes for the treatment of ALI/ARDS

**DOI:** 10.3389/fimmu.2022.971189

**Published:** 2022-08-09

**Authors:** Chang Liu, Kun Xiao, Lixin Xie

**Affiliations:** ^1^ School of Medicine, Nankai University, Tianjin, China; ^2^ Center of Pulmonary & Critical Care Medicine, Chinese People’s Liberation Army (PLA) General Hospital, Beijing, China; ^3^ Medical School of Chinese People’s Liberation Army (PLA), Beijing, China

**Keywords:** exosomes, acute lung injury, acute respiratory distress syndrome, treatment, inflammation

## Abstract

Acute lung injury (ALI)/acute respiratory distress syndrome (ARDS) is a critical clinical syndrome with high morbidity and mortality. Currently, the primary treatment for ALI/ARDS is mainly symptomatic therapy such as mechanical ventilation and fluid management. Due to the lack of effective treatment strategies, most ALI/ARDS patients face a poor prognosis. The discovery of exosomes has created a promising prospect for the treatment of ALI/ARDS. Exosomes can exert anti-inflammatory effects, inhibit apoptosis, and promote cell regeneration. The microRNA contained in exosomes can participate in intercellular communication and play an immunomodulatory role in ALI/ARDS disease models. This review discusses the possible mechanisms of exosomes in ALI/ARDS to facilitate the development of innovative treatments for ALI/ARDS.

## 1 Introduction

Acute lung injury (ALI)/acute respiratory distress syndrome (ARDS) is a severe clinical syndrome with high incidence and mortality (1). ALI/ARDS is mainly characterized by decreased lung compliance, severe hypoxemia, and progressive hypoxic respiratory failure (2). The main imaging characteristics of ALI/ARDS are diffuse, bilateral exudative lesions in the lungs (3), while the main pathophysiological features are increased pulmonary vascular permeability, pulmonary interstitial edema, fibrin exudation in alveoli, increased intrapulmonary shunt, and ventilation-perfusion imbalance (4). Epithelial injury is a marker of ALI/ARDS. The primary pathological features of ALI/ARDS are diffuse injury of alveolar epithelial cells and pulmonary capillary endothelial cells (5, 6). ALI is defined as the acute onset of diffuse bilateral pulmonary infiltrates by chest radiograph, and with a PaO2/FiO2 ≤ 300mmHg or without clinical evidence of left atrial hypertension (7). Those with more severe hypoxemia (PaO2/FiO2 ≤ 200mmHg) are considered to have ARDS. This nomenclature formulated by international consensus has been in place for nearly a decade with wide acceptance.

According to the Berlin definition, ARDS is divided into three categories in terms of the degree of hypoxemia: mild (PaO_2_/FiO_2_ = 200–300 mmHg); moderate (PaO_2_/FiO_2_ = 100–200 mmHg); and severe (PaO_2_/FiO_2_ < 100 mmHg) (8). The complex etiology of ALI/ARDS includes direct factors such as severe pneumonia, inhalation lung injury, pulmonary contusion, and ischemia-reperfusion injury along with indirect factors such as blood transfusion, acute pancreatitis, and septicemia (9). Excessive inflammatory response and the destruction of the pulmonary microvascular barrier caused by increases in endothelial and epithelial permeability are the key mechanisms involved in the pathogenesis of ALI/ARDS; as a result, ALI/ARDS is a global problem that seriously threatens human health (10).

At present, there is no specific treatment for ALI/ARDS. The clinical therapies applied in ALI/ARDS mainly include pulmonary protective ventilation and limited fluid management supplemented by glucocorticoids, inhaled pulmonary vasodilators, neuromuscular block, and extracorporeal membrane oxygenation (11, 12). While mechanical ventilation is an important treatment for ALI/ARDS, it may lead to ventilator-associated events and even pulmonary fibrosis (13–15). Restrictive fluid management can significantly improve oxygenation and shorten the duration of mechanical ventilation, but it has no significant effect on mortality. Thus, although supportive therapy can improve ALI/ARDS symptoms, it is unlikely to improve the prognosis. The mortality of patients with ALI/ARDS remains high, with the mortality of patients with severe ALI/ARDS reaching 46.1% (16). In addition, some patients with ALI/ARDS experience reduced quality of life after hospital discharge along with long-term sequelae, including cognitive impairment, psychological illness, pulmonary insufficiency, and neuromuscular weakness (17). Therefore, safe and effective therapeutic strategies for ALI/ARDS are urgently needed.

Exosomes have exhibited application prospects in a variety of diseases. Exosomes can promote tissue repair and angiogenesis to facilitate the reconstruction of ischemic tissues (18–20). Exosomes also have potential roles in the inflammatory response and autoimmune disease (21, 22). These characteristics suggest that exosomes might be applied in novel therapeutic strategies to improve the outcomes of ALI/ARDS (23–25).

## 2 Opportunities for exosome therapy in ALI/ARDS

### 2.1 Formation and main features of exosomes

According to the latest definition of the International Society of Extracellular Vesicles (ISEV), EVs are classified based on (1). physical characteristics: such as density (low, middle, high, with each range defined), or size [“small EVs” (<200 nm) and “medium/large EVs” (>200nm)]; (2). biochemical composition: (e.g., CD63^+^/CD81^+^-EVs, Annexin A5-stained EVs), and (3). cell of origin: podocyte EVs, hypoxic EVs, large oncosomes, apoptotic bodies (26).

Generally, EVs are classified into three categories based on their biogenesis and size. (1). exosomes are formed by the inward budding of the endosomal membrane and released through exocytosis (30-150nm in diameter); (2). microvesicles (MVs) are released by the directly budding of the plasma membrane (100-1000nm in diameter); and (3). apoptotic bodies are released through plasma membrane budding during programmed cell death or apoptosis. (50-5000nm in diameter) (27–30). Ultracentrifugation (UCF) is the most commonly used method for EV isolation. The centrifugation speed for exosomes is 100000-20000g (31, 32). However, UCF is time-consuming, limiting its application in the large-scale purification of EVs (28). A combination of UCF and density gradient centrifugation can be used to isolate exosomes at a relatively low density from EVs (33). Immunoaffinity capture, which depends on the presence of specific known surface protein markers of different EV types, can be used to select the required EV population through antigen-antibody correlation, ligand-receptor interaction, or magnetic techniques (34, 35). While this method is more efficient than UCF in capturing exosomes, it requires skilled operators. Emerging methods for EV separation include atomic force microscope-infrared spectroscopy, which can be used to probe the structural composition of a single EV (36). Asymmetric flow field-flow fractionation can be used to separate EVs by size at high resolution (37). Finally, nanoparticle tracking assay (NTA), transmission electron microscopy (TEM), and Western blotting can be used for the post-isolation characterization of exosomes (38). These methods above contribute to our understanding of EV biology and may be applied in the development of disease diagnosis and EV therapies.

**Figure 1:**
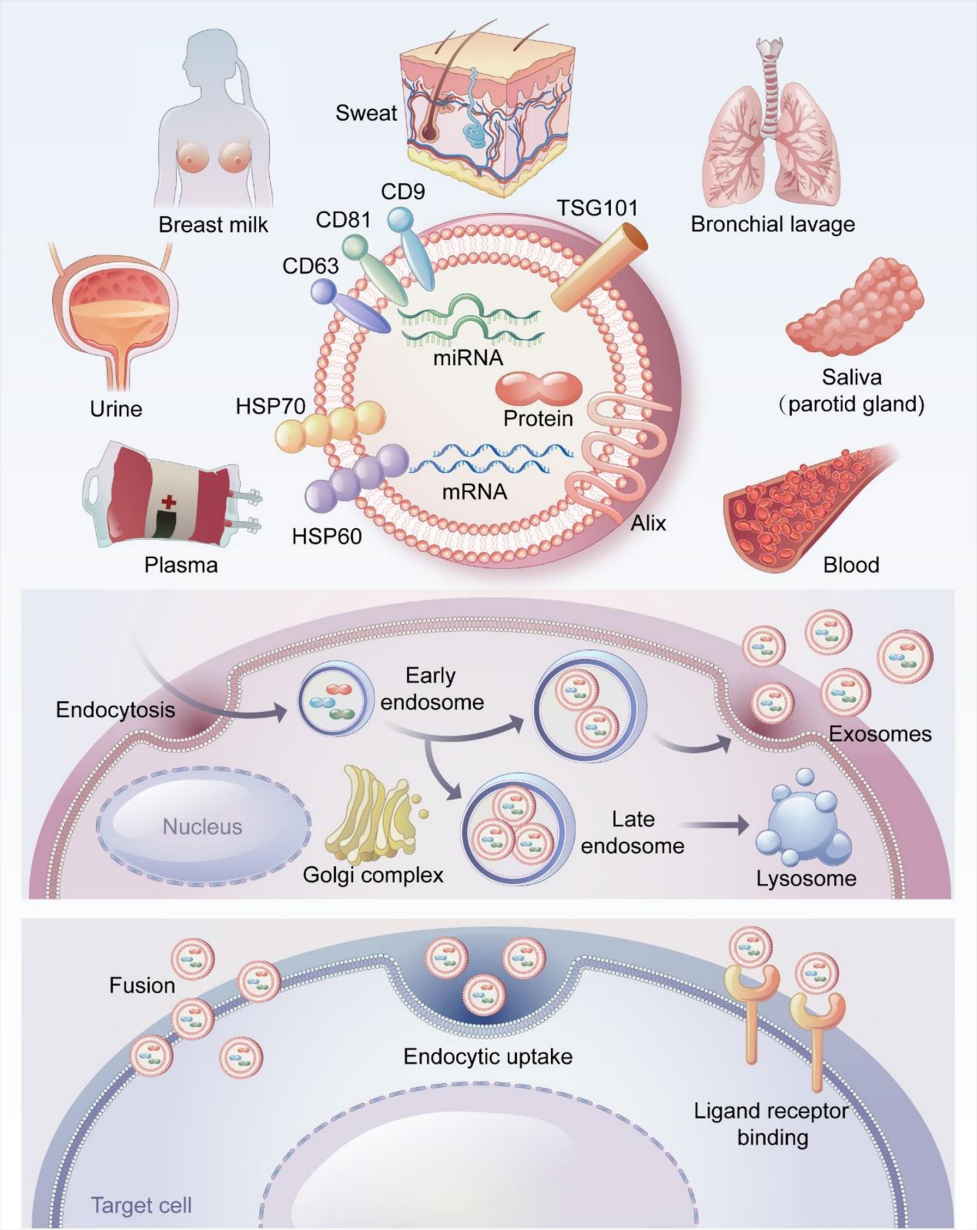
The source, formation process and surface markers of exosomes and the way of entering cells. Exosomes are derived from a variety of body fluids, such as saliva, breast milk, bronchoalveolar lavage fluid, urine, sweat, blood and plasma. CD9, CD63, CD81, TSG101, Alix and HSP70 are the most common exosome surface-specific protein markers. Early endosomes germinate to form multivesicular bodies containing multiple luminal vesicles, the binding of multivesicular bodies to lysosomes results in material degradation. In contrast, fusion with the cytoplasmic membrane leads to the release of intraluminal vesicles outside the cell. These intraluminal vesicles are called exosomes. Exosomes can directly fuse with the cell membrane of target cells, or uptaken by target cells through endocytosis. In addition, exosomes can also bind to specific receptors on the surface of target cells.

Exosomes were first found in sheep reticulocytes in 1983, when they were generally considered to be waste discharged by cells (39). Johnstone coined the term “exosomes” in 1987 (40). Exosomes are extracellular lipid bilayer vesicles with diameters of 40–100 nm and densities of 1.10–1.18 g/ml (41). Exosomes exist in a variety of body fluids including saliva (42), breast milk (43), bronchoalveolar lavage fluid (44), urine (45), sweat (46), blood, and plasma (47, 48). (**Figure 1**).

Exosomes are produced through the endosome pathway as follows (**Figure 1**). Cells germinate inward to produce early endosomes, which interact with the Golgi complex to form late endosomes. The late endosomes fuse to form multivesicular bodies, which fuse with the plasma membrane and release exosomes through exocytosis (29, 49). All cell types, including epithelial cells, tumor cells, stem cells, and immune cells, can release exosomes (50–53). Exosomes act on target cells in three ways (29, 54) (**Figure 1**): (1) directly by fusing with the cell membranes of the target cells; (2) uptake by target cells through endocytosis; and (3) binding to specific receptors on the surfaces of target cells.

Exosomes and MVs regulate normal physiological and abnormal pathological processes through basic biological functions (55). Exosomes can serve as carriers to transport proteins, lipids, and nucleic acids (mRNA, miRNA, etc.) to recipient cells, where these molecules can exert biological effects and participate in cell communication (56–59). Exosomes can also participate in immune regulation through antigen presentation and the transport of major histocompatibility complexes (60–62). CD9, CD63, CD81, TSG101 and Alix are specific markers of exosomes and participate in the formation of exosomes (63, 64) (**Figure 1**). Exosomes play a critical role in disease diagnosis, tumor-targeting therapy, and tissue repair and show great potential in clinical diagnosis and treatment (20, 65, 66).

Exosomes have emerged as a promising new therapeutic tool for cell-free therapy due to their role in tissue homeostasis. Exosomes can participate in intercellular communication and be used as an alternative to stem cells for the treatment of various diseases to promote tissue regeneration and maintain organ homeostasis after injury (67, 68). In addition, the ability of exosomes to penetrate cell membranes and target specific tissues or cells allows them to be used as carriers for specific drugs (69). Moreover, exosomes are not rejected by the immune system due to their low immunogenicity. Our review provides insights into the repair of exosomes in ALI, and in the future, once the nature and biological properties of exosomes are better understood, exosomes may become therapeutic targets for various diseases (70, 71). Finally, for the studies included in this review, the authors used NTA to evaluate the size distribution of isolated exosomes. In addition, TEM was used to analyze the morphologies of exosomes and Western blotting was used to assess the expression of exosome-specific markers (CD9, CD63, and CD81).

### 2.2 Anti-inflammatory effects of exosomes

**Figure 2:**
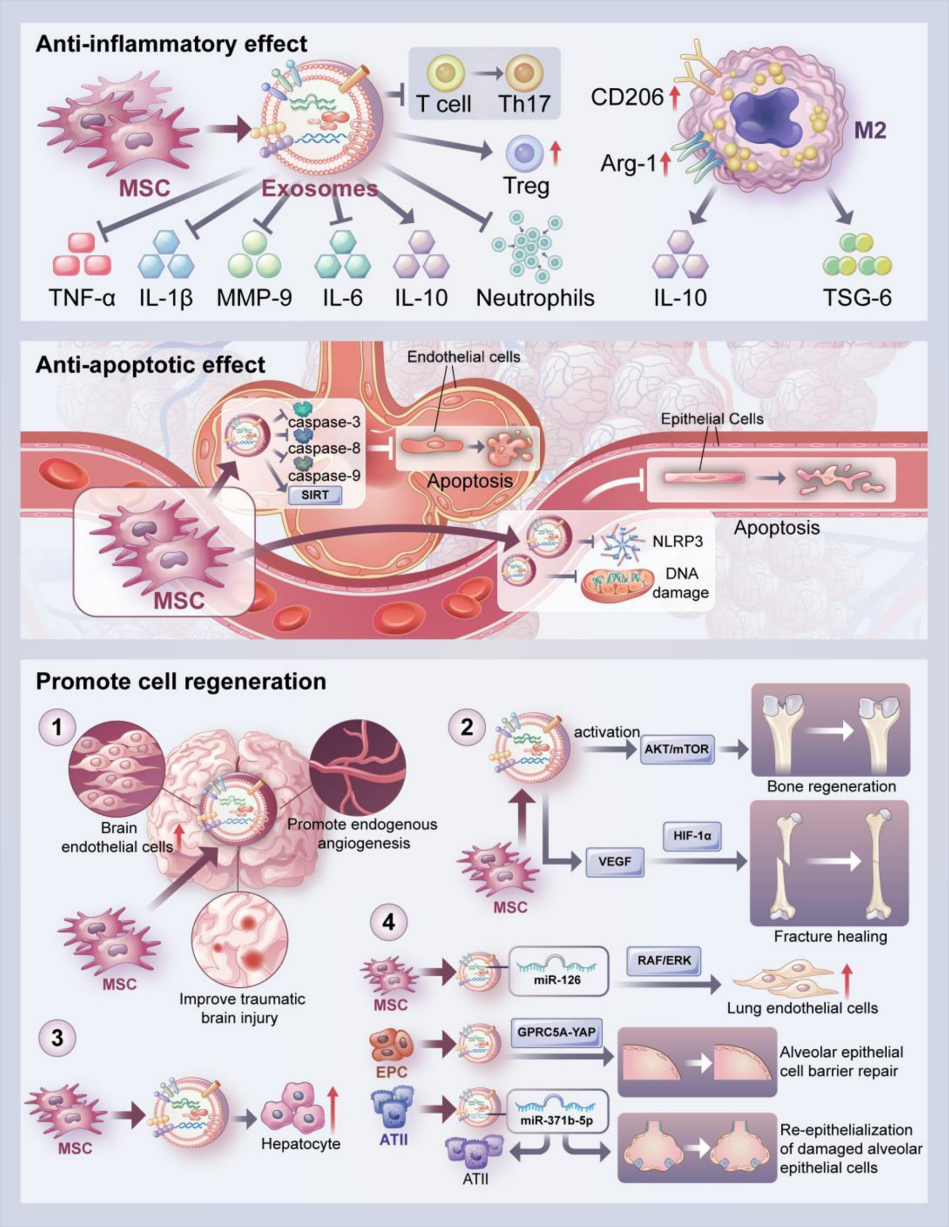
Biological effects of exosomes. The biological effects of exosomes include anti-inflammatory, anti-apoptotic and cell regeneration promoting effects. The anti-inflammatory effect of exosomes is reflected in the inhibition of the secretion of inflammatory cytokines such as TNF-α, IL-1β, IL-6, and MMP-9 and increased secretion of anti-inflammatory cytokines such as IL-10. In addition, exosomes can upregulate the expression of M2 macrophage markers such as CD206 and Arg-1, and then promote enhanced secretion of IL-10 and TSG-6 by macrophages. Moreover, MSCs-derived exosomes can inhibit the differentiation of T cells to Th17 cells and increase the level of Treg cells. They can also reduce neutrophil aggregation. Besides, exosomes can improve apoptosis of alveolar endothelial cells by inhibiting the expression of caspase-3, 8 and 9. MSCs-derived exosomes can also inhibit apoptosis of alveolar epithelial cells by inhibiting mitochondrial DNA damage and the activation of NLRP3 inflammasome, or by upregulating SIRT1 expression. Lastly, The pro-regenerative effects of exosomes were demonstrated promoting endogenous angiogenesis; promoting bone regeneration through activation of AKT/mTOR pathway and promoting fracture healing through HIF-1α-mediated angiogenesis. In addition, exosomes promoted hepatocyte proliferation, lung endothelial cell proliferation and alveolar epithelial cell barrier repair as well as the survival and proliferation of ATII cells and facilitate the re-epithelialization of damaged alveolar epithelial cells. These are the effects exosomes play in the in vitro and in vivo models.

The inflammatory disorder is a key feature of ALI/ARDS. The activation of pulmonary inflammatory response causes the recruitment of numerous immune cells along with the activation and release of cytokines, which damage pulmonary epithelial cells and increase the permeability of pulmonary microvascular endothelial cells. Meanwhile, the fluid in the alveolar cavity exudes to generate pulmonary edema, which further develops into ARDS (72, 73). Exosomes play an important role in fighting the inflammation associated with ALI/ARDS (**Figure 2**). Injecting ischemia-pretreated mesenchymal stem cell (MSCs)-derived exosomes into endotoxin-stimulated ALI mice models significantly reduced the levels of neutrophils and macrophage inflammatory protein-2 (MIP-2) (74). In an *in vitro* study, MSC-derived exosomes inhibited the secretion of pro-inflammatory factors TNF-α and interleukin (IL)-1β, increased the concentration of the anti-inflammatory factor transforming growth factor-β (TGF-β), suppressed the differentiation of T cells into Th17 cells, and increased the level of regulatory T cells (75). MSCs-derived exosomes also reduced the levels of tumor necrosis factor-α (TNF-α), IL-1β, IL-6, and matrix metalloproteinase-9 (MMP-9) and increased the level of IL-10 to modulate inflammation in phosgene-induced ALI (76). Exosomes from endothelial progenitor cells significantly decreased the number of inflammatory cells, protein concentration, expression of cytokines/chemokines, myeloperoxidase (MPO) activity, and lung injury score in bronchoalveolar lavage fluid, demonstrating their protective effect in LPS-induced lung injury (77). In addition, exosomes derived from endothelial progenitor cells decreased target genes associated with ALI such as phosphoinositide-3-kinase regulatory subunit 2 (PIK3R2) (77). Heo et al. reported that MSCs-derived exosomes could modulate macrophage polarization by increasing M2 macrophage marker expression (78). These effects might be through the activation of Stat6 and MafB transcription factors. The MSCs-induced macrophages have immunosuppressive and anti-inflammatory effects, which was achieved by high levels of anti-inflammatory cytokines IL-10 and tumor necrosis factor-stimulated gene 6 (TSG-6) (78). Wang et al. found that MSCs-derived exosomes could inhibit the aggregation of pulmonary macrophages and suppressed the synthesis and release of IL-27, and decrease the contents of IL-6, TNF-α, and IL-1β, thus alleviating sepsis-induced lung injury (79). Injection of recombinant IL-27 reversed the protective effect of exosomes on sepsis-induced lung injury. However, the authors did not investigate the downstream signaling pathway of MSCs-derived exosomes in sepsis-induced ALI. MSC-EVs significantly reduced the replication of influenza virus in the lungs along with the virus-induced production of pro-inflammatory cytokines in the lungs of influenza-infected pigs (80). However, no further identification of EVs was made in that study.

### 2.3 Anti-apoptotic effects of exosomes

Exosomes can regulate the progress of apoptosis in a variety of disease models (**Figure 2**). For example, Zhou et al. demonstrated that MSCs-derived exosomes attenuated oxidative stress and cell apoptosis by inhibiting the expression of caspase 3 and promoting cell proliferation to repair cisplatin-induced acute kidney injury (81). In addition, MSCs-derived exosomes play a protective role in lung injury by inhibiting apoptosis of alveolar epithelial cells and endothelial cells. MSCs-derived exosomes can inhibit both intrinsic and extrinsic apoptosis; the specific effect depends on the anti-apoptotic properties of miR-21-5p in the exosomes, miR-21-5p acts to reduce apoptosis in lung cells by targeting pro-apoptotic genes such as PTEN and PDCD4 and improves ischemia/reperfusion-induced lung injury. The pretreatment of MSCs with miR-21-5p antagonist completely reversed the exosome-mediated inhibition of caspase-3, -8, and -9, resulting in the persistent apoptosis of pulmonary endothelial cells (82). Sun et al. reported that exosomes improved mitochondrial DNA (mtDNA) damage and alveolar epithelial cell apoptosis by regulating the expressions of apoptosis-related proteins and mtDNA damage makers *via* the NOD-like receptor protein 3 (NLRP3) pathway, and alleviated bleomycin-induced lung injury (83). Sui et al. found that LncRNA-pretreated MSC-derived exosomes suppressed epithelial apoptosis and attenuated LPS-induced lung tissue injury by upregulating sirtuin 1 (SIRT1) expression (84). Exosomes from adipose-derived stem cells promoted autophagy, resulting in decreasing alveolar epithelial cell apoptosis and inflammatory factor expression, thus suppressing sepsis-induced lung injury (85).

### 2.4 Promotion of cell regeneration by exosomes

The role of exosomes in promoting cell regeneration has been confirmed in a variety of disease models (**Figure 2**). For example, MSCs-generated exosomes significantly improved traumatic brain injury by enhancing the number of newborn endothelial cells, promoting endogenous angiogenesis and neurogenesis, and reducing the neuroinflammation response (86). Liang et al. reported that MSCs-derived exosomes stimulated angiogenesis and bone regeneration by activating the AKT/mTOR pathway (87). Exosomes can promote angiogenesis, induce vascular endothelial growth factor (VEGF) expression, and accelerate fracture healing which was accomplished through the hypoxia-inducible factor 1α (HIF-1α) pathway (88). Tan et al. also found that MSCs-derived exosomes exhibited hepatoprotective effects to alleviate carbon tetrachloride-induced liver injury, as indicated by increased hepatocyte proliferation, enhanced Bcl-xL protein expression, and elevated cell nuclear antigen proliferation (89).

The main feature of ALI is the disruption of the alveolar and vascular membrane barrier, and repairing the barrier function to reduce alveolar epithelial-endothelial cell membrane permeability is considered as a therapeutic strategy for ALI (3). And exosomes can play an important protective role in lung injury by promoting the regeneration of alveolar epithelial cells and endothelial cells. Dinh et al. reported that lung spheroid cell-derived exosomes attenuated bleomycin-induced fibrosis by reestablishing the normal alveolar structure, indicating the therapeutic potential for lung regeneration (90). Wu et al. found that endothelial progenitor cell-derived exosomes promoted the proliferation and migration of endothelial cells and improved endothelial cell function to reduce LPS-induced pulmonary inflammation (91). Telocyte-derived exosomes ameliorated the severity of LPS-induced pulmonary edema, nourished airway epithelial cells, increased epithelial cell proliferation, and enhanced the expressions of protein kinase B (AKT), HIF-1α, and VEGF-A proteins in human bronchial epithelial cells (92). In a sulfur mustard-induced lung injury model, exosomes promoted the expressions of junction proteins, and facilitated alveolar epithelial barrier repair (93). Quan et al. demonstrated that exosomes from type II alveolar epithelial cells (ATII cells) promoted the survival and proliferation of ATII cells and promoted the re-epithelialization of damaged alveolar epithelial cells in response to bleomycin-induced lung injury (94).

## 3 Regulatory mechanism of exosomes in ALI/ARDS

### 3.1 Exosomes contribute to pulmonary protection by transferring miRNA

As a carrier of miRNA, exosomes can transfer miRNA to the corresponding receptor cells, where miRNA participates in intercellular communication, affects the lung microenvironment, and regulates the pathological process of lung diseases (95). The miRNA transmitted by exosomes can regulate ALI/ARDS by acting on related targets. MiR-126 carried by exosomes can be transported to endothelial cells, where it targets and downregulates the Sprouty-related EVH1 domain-containing protein 1 (SPRED1) to improve the function of endothelial cells and reduce LPS-induced pulmonary inflammation (91). MSCs-exosomal miR-30b-3p conferred a protective effect against LPS-induced ALI by inhibiting serum amyloid antigen 3 (SAA3) and increasing cell proliferation (96). Liu et al. showed that exosomes reduced the TNF-α, IL-1β, and IL-6 levels in a burn-induced lung injury rat model; this effect was achieved by miR-451 *via* suppressing the toll-like receptor 4 (TLR4)/NF-KB signaling pathway (97). Exosomal miR-132-3P ameliorated LPS-induced ALI/ARDS by targeting TNF receptor-associated factor 6 (TRAF6) and increasing the proliferation of mouse lung epithelial cells (98). Our team verified that MSCs-derived exosomes transmitted miR-23a-3p and miR-182-5p, which inhibited the expressions of Ikbkb and IKKβ to reverse the progression of LPS-induced ALI and advanced fibrosis (99).

MiRNA carried by exosomes can have a protective effect on ALI/ARDS by regulating macrophage function. MiR-16-5p transmitted by the exosomes of adipose mesenchymal stem cells can ameliorate lung injury in cecal ligation and puncture-induced septic mice by inhibiting TLR4 to promote anti-inflammatory M2 polarization, as evidenced by the increased expressions of specific surface markers of M2 macrophages (Arg-1 and CD206) (100). Song et al. demonstrated that the expression of miR-146a was significantly enhanced in IL-1β-pretreated MSCs-derived exosomes compared with the MSCs-derived exosomes group without IL-1β treatment, and exosomal miR-146a transferring to macrophages led to M2 polarization (101). Song et al. also reported that the transfection of miR-146a inhibitors partly counteracted the immunomodulatory properties of exosomal miR-146a (81). Autophagy is an important mechanism for maintaining the homeostasis of the internal environment, and the dysfunction of autophagy often leads to cellular injury (102). MSCs-derived exosomes can transfer miR-384-5p to alveolar macrophages and attenuate autophagic stress in alveolar macrophages through the downregulation of Beclin-1, thereby reducing the severity of LPS-induced ALI (103).

Due to partial overlap between the size of exosomes and MVs, the resulting samples may not be a highly pure population of exosomes. MVs may also play a repair role in lung injury. MVs can reduce lung injury and inflammatory response by secreting keratinocyte growth factor (KGF), and could enhance macrophage phagocytic activity and bacterial clearance, thus improving the survival rate of *Escherichia coli* endotoxin-induced lung-injured mice (104, 105). In addition, MVs can also increase alveolar fluid clearance and decrease lung protein permeability, potentially by transferring KGF mRNA to target cells (106). CD44 receptors may play a role in the entry of MVs into damaged cells to exert their effects (107).

MVs also show the therapeutic potential in ARDS through a unique miRNA transfer mechanism. For instance, MVs can inhibit macrophage activation and reduce *Klebsiella pneumoniae*-induced lung inflammation by delivering miR-223/142 to inhibit the activation of the NLRP3 inflammasome (108). MSC-MVs can exert therapeutic effects in bleomycin-induced ALI by transferring miR-100, which targets the mechanistic target of rapamycin (mTOR) to enhance autophagy (109). Future studies will further elucidate the role of exosomal miRNA in the pathogenesis of lung diseases and provide more guidance for corresponding therapeutic approaches.

### 3.2 Exosomes exert a pulmonary protective effect through a variety of signal transduction pathways

#### 3.2.1 Exosomes contribute to pulmonary protection through the MAPK signaling pathway

The mitogen-activated protein kinase (MAPK) signaling pathway is important in eukaryotes and can be activated by a variety of extracellular and intracellular signals or stimuli such as cytokines, hormones, and various stressors (110). As the MAPK signaling pathway plays a key role in cell growth, migration, proliferation, differentiation, and apoptosis, this pathway is involved in the pathogenesis of human diseases including ALI/ARDS (111, 112).

Xu et al. reported that bone marrow MSCs-derived exosomes from mice attenuated LPS-induced pulmonary microvascular endothelial cell injury and lung permeability, inhibited inflammatory factor expression, and reduced the infiltration of inflammatory cells such as neutrophils and macrophages, which was mediated by exosomal miR-150 (113). The authors also observed that the expression of MAPK pathway proteins was significantly lower in the exosome group than in the LPS group, suggesting that exosomes can reduce LPS-induced lung injury by regulating the MAPK pathway. Thus, the regulation of the MAPK pathway is a potential therapeutic mechanism for exosomes in lung injury.

#### 3.2.2 Exosomes participate in pulmonary protection through the NF-kB signaling pathway

The NF-kB signaling pathway involves a family of key transcription factors that participate in immune regulation and inflammatory response. This signaling pathway plays a critical role in maintaining normal host physiology. A variety of stimuli such as infection and the presence of pro-inflammatory cytokines can activate NF-kB. Once NF-kB is activated through phosphorylation, it induces the production of numerous inflammatory cytokines including TNF-α, IL-1β, and IL-6 (114, 115). In an *Escherichia coli* endotoxin-induced ALI model, the activation of the NF-kB pathway was found to promote the pulmonary inflammatory response, and sustained NF-kB activation was correlated with the severity of lung injury (116). Xu et al. reported that treatment with MSCs-derived exosomes attenuated NF-kB activation and reduced the expressions of pro-inflammatory cytokines, thereby inhibiting the inflammatory response and apoptosis (117). These results suggest that the NF-kB pathway is a potential target for MSCs-derived exosomes acting to protect the lungs from smoke inhalation-induced acute injury. Under LPS stimulation, the NF-kB pathway initiated the transcription of inflammatory mediators and chemokines. Enhanced miR-22-3p expression reduced oxidative stress response and suppressed apoptosis while significantly reducing the protein level of p-NF-kB, thereby reversing LPS-induced ALI (118).

TLR4 is an important pattern recognition receptor that recognizes extracellular stimuli, initiates downstream signaling pathways, activates the NF-kB pathway through the myeloid differentiation protein 88 (Myd88) non-dependent pathway and the Myd88-dependent pathway, and upregulates the expressions of various inflammatory factors (119, 120). The administration of MSCs-derived exosomes had a significant anti-inflammatory effect in severe burn-induced ALI and attenuated the expressions of proteins related to the TLR4/NF-kB pathway; these effects were reversed when the miR-451 expression in the exosomes was inhibited (97). Moreover, in a preclinical model of ALI resulting from intestinal ischemia-reperfusion, the injection of MSCs-derived exosomes reduced alveolar and interstitial edema, pulmonary hemorrhage, and inflammatory cell infiltration while downregulating the expressions of TLR4 and NF-kB, thereby reducing apoptosis in lung tissue (121). Thus, MSCs-derived exosomes provide a potential therapeutic strategy for ALI.

#### 3.2.3 Exosomes contribute to pulmonary protection through the PI3K-AKT signaling pathway

The phosphatidylinositol 3-kinase **(**PI3K)-AKT signaling pathway is an important intracellular signaling pathway that is involved in biological processes such as cell proliferation and survival, apoptosis, angiogenesis, protein synthesis, and lipid metabolism (122, 123). The PI3K-AKT signaling pathway participates in the pathophysiological processes of many diseases, including ALI/ARDS (124, 125).

Exosomal miR-126 derived from adipose-derived MSCs can attenuate histone-induced endothelial cell apoptosis and reduce pulmonary vascular permeability by activating the PI3K-AKT signaling pathway. In contrast, the administration of an AKT inhibitor increased histone-induced vascular leakage (126). Moreover, in a model of hyperoxia-induced ALI, exosomes derived from MSCs ameliorated the decrease in PI3K and AKT phosphorylation caused by oxidative damage in rat lung tissue by activating the PI3K/AKT signaling pathway, thereby reducing the extent of oxidative stress and pathological injury in the lung tissue (127).

The *in vitro* treatment of MSCs with lung-derived exosomes isolated from rats with ALI after phosgene exposure promoted the secretion of the anti-inflammatory factor IL-10 and paracrine cytokines VEGF and HGF and increased the immunomodulatory effect of the MSCs, which was partially achieved *via* miR-28-5p (128). While the activation of the PI3K/AKT signaling pathway was detected in this process, further *in vivo* studies are needed to verify whether lung-derived exosomes play a role in ameliorating lung histopathological changes during ALI. Moreover, some studies have produced conflicting results. For example, Liu et al. reported that MSC-derived exosomes attenuated LPS-induced ALI and alveolar epithelial cell apoptosis in mice by inactivating the PI3K/AKT signaling pathway (98).

#### 3.2.4 Exosomes contribute to pulmonary protection through the Hippo-YAP signaling pathway

The Hippo/YAP signaling pathway regulates cell proliferation and organ regeneration and promotes pulmonary vascular barrier repair along with lung inflammation remission and alveolar regeneration after alveolar epithelial cell injury (129–131). Exosomes derived from bone marrow-derived MSCs promoted the expressions of anti-apoptotic proteins and ligand proteins by activating the Hippo-YAP pathway, reduced lung parenchymal congestion and alveolar epithelial cell apoptosis, decreased inflammatory response, and promoted pulmonary epithelial barrier repair, resulting in a protective effect against Sulfur mustard-lung injury (93).

#### 3.2.5 Exosomes contribute to pulmonary protection through the STAT3 signaling pathway

The signal transducer and activator of the transcription 3 (STAT3) signaling pathway is activated by a variety of cytokines and growth factors and participates in a variety of biological processes such as cell proliferation, survival, differentiation, and angiogenesis (132). The role of the STAT3 signaling pathway in lung inflammation is controversial. Some studies have found that inhibiting the STAT3 signaling pathway reduces the levels of inflammatory factors in lung tissue and attenuates lung injury (133, 134). In contrast, Jiang et al. found that miR-43c-3p expression was decreased in MSCs treated with lung-derived exosomes from phosgene-induced ALI rats, thereby promoting the anti-inflammatory and proliferative properties of the MSCs by activating the JAK1-STAT3 signaling pathway to attenuate phosgene-induced ALI (135). Therefore, targeting STAT3 is a potential strategy for the treatment of lung injury.

In summary, exosomes can exert a pulmonary protective effect through various signaling pathways such as NF-kB, MAPK, PI3K-AKT, Hippo-YAP and STAT3 signaling pathway. The signaling pathways involved may have complex effects. And these effects of exosomes may depend on their contents, sources and disease models.

### 3.3 Exosomes contribute to pulmonary protection by participating in mitochondrial transfer and repairing mitochondrial dysfunction

In the occurrence and development of ALI, reactive oxygen species produced by the mitochondria lead to mitochondrial DNA transcription defects and mitochondrial dysfunction. Damaged mitochondrial DNA can further drive adverse reactions in the lungs (136, 137). Hough et al. confirmed that the exosomes of airway myeloid-derived regulatory cells improved the T cell response in chronic inflammation by transferring mitochondria to T cells (138). This process may play a role in acute inflammation disorders such as ALI, although additional research is required. MSCs-derived exosomes ameliorated the production of reactive oxygen species and mitochondrial DNA damage induced by bleomycin to reduce the apoptosis of alveolar epithelial cells (83). MiR-Let-7, which was encapsulated in the exosomes, played an important role by regulating the expression of lectin-like oxidized low-density lipoprotein receptor-1 (LOX1); meanwhile, the high expression of LOX1 regulated NLRP3 to promote the expression of apoptosis-related proteins and markers of mitochondrial DNA damage (63).

MSCs-derived extracellular vesicles can transfer mitochondria to alveolar macrophages, increase macrophage oxidative phosphorylation, promote macrophage polarization to an anti-inflammatory phenotype and enhance phagocytosis of macrophages, and improve LPS-induced lung injury *in vivo* (139).. Sliva et al. confirmed that LPS stimulation damaged the mitochondrial membrane potential, increased the production of mitochondrial reactive oxygen species, and inhibited mitochondrial respiration (140). The mitochondrial transfer of MSCs-derived extracellular vesicles restored normal mitophagy and mitochondrial biogenesis, alleviated LPS-inhibited mitochondrial respiration, relieved mitochondrial dysfunction, and restored the barrier integrity in alveolar epithelial cells (114). However, these studies did not further classify the extracellular vesicles contained in the MSCs. Therefore, additional studies are needed on the regulation of mitochondrial function by MSC-derived exosomes to demonstrate that these exosomes can attenuate ALI.

## 4 The safety and efficacy of exosomes in animal experiments and clinical application in ALI/ARDS

### 4.1 Effectiveness of exosomes in animal models of ALI/ARDS

MSCs-derived exosomes have shown good therapeutic effects in preclinical models of ALI/ARDS. MSCs-derived exosomes have been shown to inhibit macrophage aggregation in lung tissue, suppress IL-27 secretion, reduce the levels of pro-inflammatory factors in lung tissue, and improve the survival rate of mice (79). Wei et al. reported that human umbilical cord MSCs-derived exosomes reduced the levels of inflammatory factors and inhibited lung inflammation and oxidative stress in LPS-induced ALI by inducing autophagy *in vivo* (141).. Lastly, Tian et al. demonstrated the therapeutic effects of exosomes (primarily improved pathological injury and promoted M2 macrophage polarization) derived from adipose-derived MSCs on septic lung injury in mice (100).

In addition to mesenchymal stem cell-derived exosomes, other cell-derived exosomes such as endothelial progenitor cell (EPC), alveolar macrophage, alveolar progenitor type II cell, amniotic epithelial cell, and adipose tissue cell, also play a protective role in ALI/ARDS animal models. For example, EPC-derived exosomes have been shown to reduce LPS-induced lung injury by decreasing local inflammatory cytokines, pulmonary permeability, and neutrophil migration (77). Wu et al. reported that EPC-derived exosomes can improve ALI/ARDS prognosis by promoting the expression of the RAK/ERK signaling pathway and thus improving endothelial cell function (91). This was achieved by transferring miR-126 to endothelial cells and downregulating the expression of SPRED1. Alveolar macrophage-derived exosomes can regulate inflammatory signaling by transferring suppressors of cytokine signaling (SOCS) 1 and 3 and suppressing STAT in alveolar epithelial cells (142).

In bleomycin-induced lung injury models, exosomes from alveolar progenitor type II cells promoted the proliferation of ATII cells and enhanced epithelial regeneration in damaged alveolar cells, thereby providing a new target for the treatment of lung diseases (94). Exosomes from amniotic epithelial cells could increase macrophage phagocytosis, decrease the level of neutrophil peroxidase, inhibit T cell proliferation and reduce lung inflammation (52). Jiang et al. found that endothelial cell-derived exosomes could inhibit topoisomerase II α (TOP2A) expression, resulting in a protective role in sepsis-induced ALI (143). This effect was achieved by the transfer of miR-125b-5p. and the transfection of miR-125b-5p inhibitor reversed the protective effect of the exosomes in ALI.

Finally, adipose tissue-derived exosomes improve ventilator-induced lung injury (VILI) by reducing the permeability of the pulmonary endothelial barrier and attenuating the inflammatory response, with is potentially related to the inhibition of the transient receptor potential vanilloid 4 (TRPV4)/Ca2+ pathway. The inhibition of TRPV4 attenuated the increase in pulmonary barrier permeability and reduced the release of pro-inflammatory cytokines induced by mechanical ventilation, resulting in a protective effect against VILI (144).

### 4.2 Safety and efficacy of exosomes in clinical trials of ALI/ARDS

Mitrani et al. reported that treatment of patients with severe ARDS caused by COVID-19 with the exosome-containing agent Zofin was safe and well tolerated, with no reports of any serious adverse events (145). The Zofin treatment improved respiratory function, decreased the expressions of the inflammatory biomarkers C-reactive protein (CRP) and IL-6, and reduced the Sequential Organ Failure Assessment scores. In a clinical trial of MSCs-derived exosomes for the treatment of patients with ARDS caused by severe COVID-19, patients given intravenous bone marrow MSCs-derived exosomes showed improvement in clinical symptoms and oxygenation status, significant reductions in acute phase reactants such as CRP, ferritin, and D-dimer, and increases in absolute neutrophil counts and absolute lymphocyte counts without any adverse events (146). While the study demonstrates the safety and efficacy of exosomes in the treatment of patients with ARDS, it has some limitations. For example, the exosomes used were derived from bone marrow. However, the sources of exosomes are complex and heterogeneous. In addition to bone marrow, exosomes can also originate from the placenta, umbilical cord, and amniotic membrane. Thus, the results of the abovementioned clinical study cannot be interpreted as class effects. **Table 1** shows the results returned by a search of the ClinicalTrial.gov website for ongoing or completed clinical trials on ALI/ARDS. The findings suggest that exosomes are a promising therapeutic candidate for patients with ALI/ARDS, and more randomized controlled studies are needed to demonstrate their safety and efficacy.

**Table 1:** Summary of clinical trials involving exosomes in ALI/ARDS patients.

Nct Number		Study Title		Number Enrolled	Status	Locations	Study Completion	Phase	Exosome Sources	Study Type
NCT04602104		A Clinical Study of Mesenchymal Stem Cell Exosomes Nebulizer for the Treatment of ARDS		169	Recruiting	Ruijin Hospital, Medical School of Shanghai Jiaotong University Shanghai, Shanghai, China	September, 2022	1,2	human mesenchymal stem cell- exosomes	Interventional
NCT04798716		The Use of Exosomes for the Treatment of Acute Respiratory Distress Syndrome or Novel Coronavirus Pneumonia Caused by COVID-19		55	Not yet recruiting	Mission Community Hospital Panorama City, California, United States	December, 2024	1,2	Mesenchymal stem cell- exosomes	Interventional
NCT04747574		Evaluation of the Safety of CD24- Exosomes in Patients With COVID-19 Infection		35	Completed	Tel Aviv Medical Center Tel Aviv, Israel	Marth 25th, 2021	1	CD24- exosomes	Interventional
NCT04493242		Extracellular Vesicle Infusion Treatment for COVID-19 Associated ARDS		120	Completed	Helen Keller Hospital Sheffield, Alabama, United States St. Joseph Hospital Heritage Fullerton, California, United States Donald Guthrie Foundation/ Robert Packer Hospital Sayre, Pennsylvania, United States	May 22nd, 2021	2	Bone marrow mesenchymal stem cell EXO-FLO	Interventional
NCT05127122		Bone Marrow Mesenchymal Stem Cell Derived Extracellular Vesicles		81	Not yet recruiting	Not mentioned	August, 2022	1,2	Bone marrow mesenchymal stem cell EXO-FLO	Interventional
NCT04657458			Infusion Treatment Expanded Access Protocol on Bone Marrow Mesenchymal Stem Cell Derived Extracellular Vesicle Infusion Treatment for Patients With COVID-19 Associated ARDS	Not mentioned	Avaliable	Not mentioned	Not mentioned	2	Bone marrow mesenchymal stem cell EXO-FLO	Expand Access	
NCT04969172			A Phase II Randomized, Double-blind, Placebo- controlled Study to Evaluate the Safety and Efficacy of Exosomes Overexpressing CD24 to Prevent Clinical Deterioration in Patients With Moderate or Severe COVID-19 Infection	155	Active, not recruiting	Tel-Aviv Sourasky Medical Center Tel-Aviv, Israel	July 11th, 2022	2	Exosomes overexpressing CD24	Interventional	
NCT04276987			A Pilot Clinical Study on Inhalation of Mesenchymal Stem Cells Exosomes Treating Severe Novel Coronavirus Pneumonia	24	Completed	Ruijin Hospital Shanghai Jiao Tong University School of Medicine Shanghai, Shanghai, China	July 31st, 2021	1	MSC-derived exosomes	Interventional	

Currently, there are few Good Manufacturing Practice compliant protocols (147). Given the diversity of sources and isolation methods of MSC-EVs,validation metrics and functional analysis are needed to better characterize MSC-EVs. Strict “identity” and “efficacy” parameters such as cellular origin, the degree of physical and biochemical integrity of the vesicles, must be defined before MSC-EVs can be used in therapeutic applications (148). Since global standardization of MSC-EVs production is unlikely, there is a need to define MSC-EVs agents physically, biochemically, and functionally through quantifiable characteristics and the use of reproducible and standardized analyses.

## 5 Conclusions and future prospects

**Figure 3:**
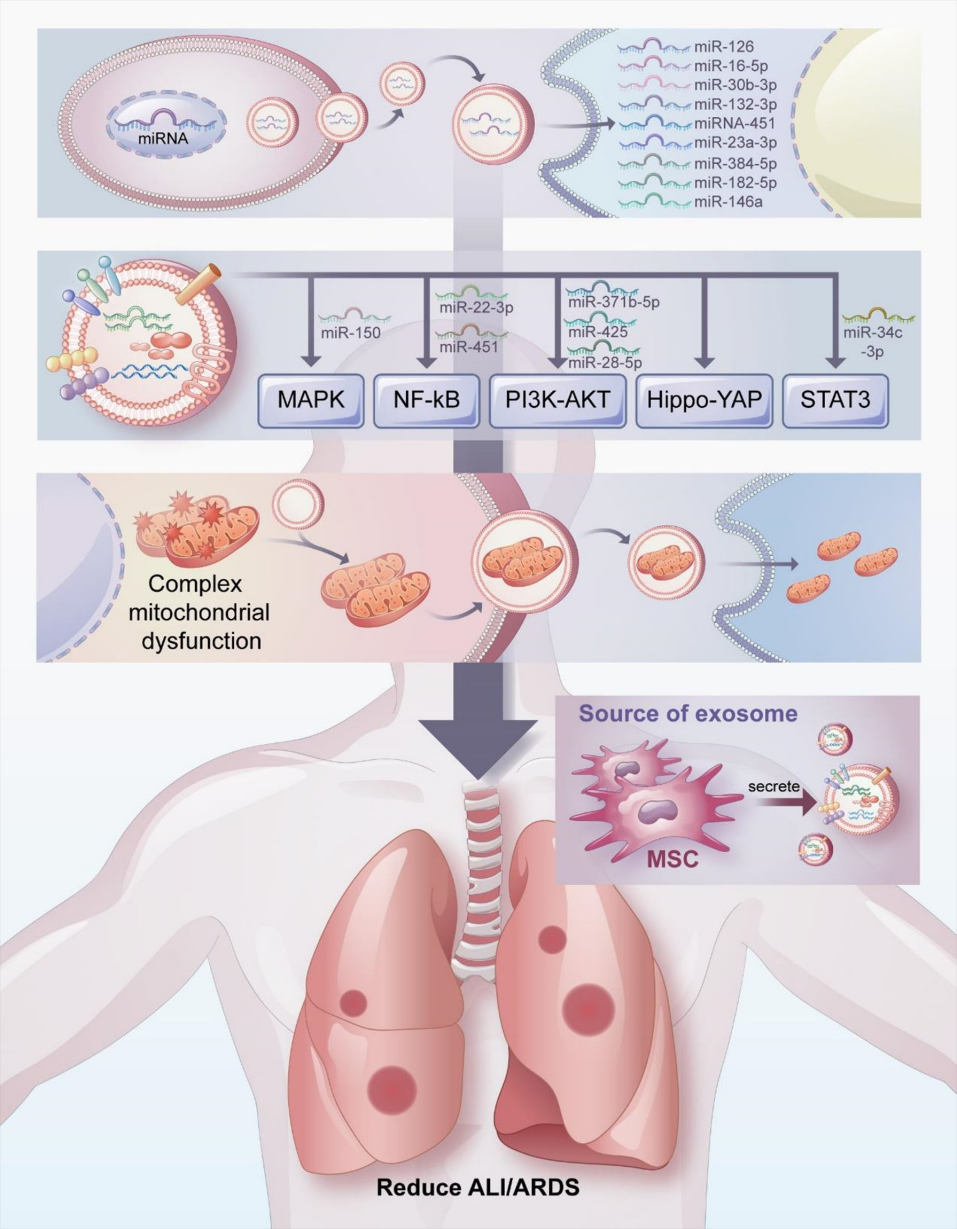
Mechanisms by which exosomes attenuate ALI/ARDS and thus exert pulmonary protective effects. The potential therapeutic mechanisms of exosomes in the treatment of ALI/ARDS include transferring miRNA, participating in signal pathway transduction, and regulating mitochondrial function.

Exosomes have attracted considerable attention as a therapeutic agent for the treatment of ALI/ARDS. In this review, we have discussed the potential therapeutic mechanism of exosomes in the treatment of ALI/ARDS, which include transferring miRNA, participating in signal pathway transduction, and regulating mitochondrial function (**Figure 3**). Exosomes have many beneficial properties (71, 149, 150). For example, the non-immunogenicity of exosomes prevents immune rejection and tumorgenicity. As a cell-free therapy, exosomes do not block the blood vessels of organs or cause embolism because they penetrate deeply into most organs. In addition, exosomes can be modified and loaded with drugs of interest, and their surface-specific receptors can be artificially processed to transfer the exosomes to target cells.

Given the above advantages, exosomes show great promise for clinical application and may become a leading treatment for ALI/ARDS in the future. However, the clinical application of exosomes remains challenging, and considerable additional research is needed to achieve this goal. First, most existing studies on exosomes for the treatment of ALI/ARDS were preclinical (mostly cellular or animal studies). Whether exosomes behave similarly in the human body remains unclear. Second, the mechanisms through which exosomes affect ALI/ARDS are not fully understood, and many mechanisms are yet to be explored. Third, determining the optimal conditions for treating ALI/ARDS with exosomes (e.g., the optimal dosage, actuation duration, and administration route) remain challenges for clinical application. Finally, considering the various sources of exosomes, the quality and therapeutic effect of exosomes will depend on the culture conditions and pretreatment methods. Thus, a quality standard for formulating exosomes that are stable and easy to store needs to be established.

In conclusion, exosomes show great potential for the treatment of ALI/ARDS and are expected to become an effective treatment option. However, numerous challenges must be addressed before exosomes can be applied in clinical medicine. There is also a lack of reports on adverse events associated with the application of exosomes. Therefore, additional research is needed to explore the mechanisms and long-term effects of exosomes in the treatment of ALI/ARDS.

## Author contributions

CL drafted the initial manuscript, KX and LX revised the manuscript. All authors contributed to the article and approved the submitted version.

## Funding

This work was supported by China PLA Scientific Key Grant (20-163-12-ZT-005-003-01), China Key Scientific Grant Program (No. 2021YFC0122500), National Science Foundation for Young Scientists of China (Grant No. 82100096) and National Science Foundation for Young Scientists of Beijing (Grant No. 7214254).

## Acknowledgments

The authors thank AiMi Academic Services (www.aimieditor.com) for the English language editing and review services.

## Conflict of interest

The authors declare that the study was conducted without any commercial or financial relationships that could be interpreted as potential conflicts of interest.

## Publisher’s note

All claims expressed in this article are solely those of the authors and do not necessarily represent those of their affiliated organizations, or those of the publisher, the editors and the reviewers. Any product that may be evaluated in this article, or claim that may be made by its manufacturer, is not guaranteed or endorsed by the publisher.
